# Tricyclazole alleviates *Fonsecaea pedrosoi*-induced immune suppression of neutrophils by inhibiting DHN-melanin biosynthesis

**DOI:** 10.3389/fcimb.2025.1697287

**Published:** 2026-01-12

**Authors:** Chunjiao Zheng, Wei Li, Yuanyuan Wang, Lulu Li, Linan Ni, Xiaoping Liu, Jingwen Tan, Lianjuan Yang, Qian Yu

**Affiliations:** 1 Department of Medical Mycology, Center of Infectious Skin Diseases, Shanghai Skin Disease Hospital, Tongji University School of Medicine, Shanghai, China; 2 Department of Medical Cosmetology, Shanghai Skin Disease Hospital, Tongji University School of Medicine, Shanghai, China

**Keywords:** chromoblastomycosis, DHN-melanin synthesis, *Fonsecaea pedrosoi*, NETs (neutrophil extracellular traps), oxidative stress, tricyclazole

## Abstract

**Introduction:**

Chromoblastomycosis (CBM) is a chronic cutaneous infection caused by dematiaceous fungi, characterized by therapeutic challenges such as difficulty in pathogen clearance and high recurrence rates. *Fonsecaea pedrosoi* (*F. pedrosoi*), the most common etiological agent of CBM, relies on its virulence factor DHN-melanin to evade host immune responses—especially by suppressing neutrophil function—further contributing to disease persistence and treatment resistance. Thus, we intended to explore therapeutic approaches that target both fungal virulence mechanisms and host immune regulation to overcome the clinical hurdles of CBM.

**Objectives:**

This study aimed to investigate the effects of tricyclazole (TCZ) on *F. pedrosoi* and neutrophil antifungal responses, with a particular focus on its potential actions in inhibiting DHN-melanin synthesis and enhancing host oxidative immune mechanisms.

**Methods:**

We conducted *in vitro* assays to assess the effects of TCZ on *F. pedrosoi* melanin and fungal antioxidant enzymes, as well as reactive oxygen species (ROS) production and neutrophil extracellular traps (NETs) formation in human neutrophils. The *in vivo* mouse model was used to evaluate inflammatory responses, neutrophil-related markers, and fungal clearance.

**Results:**

*In vitro* tests showed TCZ dose-dependently inhibited fungal DHN-melanin synthesis and disrupted the antioxidant enzyme system (including superoxide dismutase and catalase); this effect not only weakens the fungus’s ability to resist host oxidative stress but also reduces its capacity to evade immune recognition, creating conditions for subsequent immune clearance. *In vitro* co-culture models revealed that TCZ significantly enhanced neutrophil ROS production and NET formation; this strengthens the oxidative killing function of neutrophils, directly counteracting the immunosuppressive effect of DHN-melanin on neutrophils and improving the host’s ability to eliminate pathogens. In a mouse infection model, TCZ treatment significantly alleviated pedal inflammation, reduced neutrophil activation markers, and completely eliminated fungal colonization; these results validate TCZ’s *in vivo* therapeutic efficacy, demonstrating its potential to mitigate inflammatory tissue damage while achieving effective fungal eradication.

**Conclusions:**

This study reveals a novel mechanism by which TCZ counteracts *F. pedrosoi*-mediated suppression of neutrophil antifungal effector functions, particularly oxidative burst and NET formation, thereby facilitating fungal clearance in CBM. These findings provide a novel strategy for CBM treatment by integrating immunomodulation with antifungal therapy.

## 1 Introduction

Chromoblastomycosis (CBM) is a chronic, progressive cutaneous and subcutaneous infection caused by dematiaceous fungi, including *Fonsecaea pedrosoi*, *Fonsecaea compacta*, *Phialophora verrucosa*, and *Cladophialophora carrionii*, which are prevalent in tropical and subtropical regions worldwide (Queiroz-Telles et al., 2017; Queiróz et al., 2018). *Fonsecaea pedrosoi* (*F. pedrosoi*) is the most common etiological agent of CBM (Santos et al., 2007). CBM lesions are profoundly refractory and often considered nearly intractable in clinical settings, with a notable tendency for recurrence. Management of moderate to severe cases remains a persistent clinical challenge (Brito and Bittencourt, 2018). Incomplete eradication of pathogenic fungi from infected skin tissues is a primary cause of treatment failure (Brito and Bittencourt, 2018). Therefore, understanding pathogen–host interactions and identifying potential therapeutic targets is critical to improving clinical outcomes.

Over the past decade, significant efforts have been made to elucidate host immune responses in CBM. While many studies have emphasized the role of T cells and interferon-γ (IFN-γ) in disease control, innate immune responses are increasingly recognized as playing a more prominent role in CBM pathogenesis, though they remain poorly understood (Mazo Fávero Gimenes et al., 2005; Breda et al., 2020b). Previous literature has reported an abundance of macrophages and neutrophils in CBM lesion granulomas, suggesting their involvement in antigen presentation and the host immune response. Resident macrophages that ingest *F. pedrosoi* conidia may support hyphal growth, leading to macrophage death (Rozental et al., 1994; Bocca et al., 2006; Breda et al., 2020b). In contrast, IFN-γ-preactivated macrophages exhibit fungistatic activity, reducing hyphal growth and maintaining macrophage viability (Rozental et al., 1994; Bocca et al., 2006; Breda et al., 2020b). Unlike macrophages, neutrophils directly mediate pathogen killing and are recognized as highly proinflammatory cells with antimicrobial capabilities, including phagocytosis, oxidative burst, degranulation, and neutrophil extracellular trap (NET) release (Breda et al., 2020a). NETosis, a unique form of programmed neutrophil cell death, functions by releasing NETs (Hidalgo et al., 2022; Liu et al., 2025). Activated neutrophils entrap and kill pathogens by releasing NETs, which are a crucial defense mechanism during infection, injury, or inflammation (Liu et al., 2025). As early as the 1990s, Rozental’s team demonstrated that neutrophils exhibit fungicidal activity against *F. pedrosoi* conidia (Rozental et al., 1996). Additionally, neutrophils indirectly regulate infection by secreting IL-17, which recruits Th17 lymphocytes, a cell population critical in controlling fungal infections (Eskan et al., 2012; Chen et al., 2014). Overall, neutrophil activation is consistently associated with pathogen containment and elimination, highlighting the need to explore the regulation of neutrophil function in CBM.

Melanin, a key virulence factor in *F. pedrosoi*, is synthesized via oxidative polymerization of 1,8-dihydroxynaphthalene (1,8-DHN). This pigment is insoluble in water and organic solvents. Its unique hydrophobicity, free radical-scavenging ability, and metal-chelating properties allow it to effectively counteract oxidative killing by host immune cells. During *F. pedrosoi* infection, DHN-melanin neutralizes oxidative burst products such as H_2_O_2_ and NO produced by macrophages and significantly reduces neutrophil recognition and phagocytosis by masking fungal cell wall epitopes, thereby promoting fungal persistence in the host (Cunha et al., 2010; Zhang et al., 2013). However, the interaction between DHN-melanin and neutrophil immune responses remains unclear. Tricyclazole (TCZ), a melanin biosynthesis inhibitor, is widely used as a fungicidal agrochemical for managing rice blast disease (Kumar et al., 2015). Cunha’s team demonstrated that TCZ enhances macrophage-mediated phagocytic killing of *F. pedrosoi* by depleting melanin, which in turn exposes β-glucans on the fungal cell wall and facilitates recognition by macrophage surface Dectin-1 receptors (Cunha et al., 2010). Whether TCZ can modulate neutrophil function by inhibiting DHN-melanin synthesis in *F. pedrosoi* remains an important and unanswered question.

Building on the aforementioned findings, this study aimed to explore whether TCZ augments antifungal immunity through a mechanism involving DHN-melanin biosynthesis inhibition and mitigation of melanin-induced suppression of neutrophil oxidative burst and NETosis pathways. To this end, both *in vitro* experiments and *in vivo* studies in mice were conducted to examine TCZ’s effect on the biosynthesis of fungal DHN-type melanin, assess its capacity to mitigate melanin-mediated inhibition of neutrophil oxidative stress responses and NET formation, and thereby clarify the role of TCZ in restoring neutrophil antifungal functions. Our results provide compelling evidence for TCZ’s immunorestorative potential and suggest a novel therapeutic strategy that synergistically combines melanin biosynthesis inhibition with neutrophil functional enhancement to combat refractory fungal infections.

## 2 Materials and methods

### 2.1 Experimental animals

Female BALB/c mice (6–8 weeks) were purchased from Shanghai Jiesijie Laboratory Animal Co., Ltd. The mice were housed in a specific pathogen-free facility under a 12-hour light/dark cycle, with controlled environmental conditions (temperature: 22 ± 2°C; relative humidity: 40–60%). Standard rodent maintenance feed and sterilized drinking water were provided. Mice were allowed to acclimate to the environment for 1 week before experimentation. All animal protocols were approved by the Institutional Animal Ethics Committee of Shanghai Dermatology Hospital.

### 2.2 Experimental reagents

#### 2.2.1 Cell and immunology-related reagents

Human peripheral blood neutrophils were obtained from Yuancreative Biotechnology Co., Ltd. (Shanghai, China). Fluorescein isothiocyanate (FITC) from Sigma-Aldrich. Anti-CD45 antibody, Anti-neutrophil elastase antibody, anti-histone H3 (citrulline R2+R8+R17) antibody, and goat anti-rabbit IgG H&L were purchased from Abcam. Anti-human myeloperoxidase (MPO), anti-histone H3 antibody, and anti-neutrophil antibody were purchased from Thermo Fisher Scientific. QuickBlock™ blocking buffer was obtained from Beyotime (Shanghai, China). Tricyclazole and DNase I were acquired from Shanghai Aladdin Biochemical Technology Co., Ltd.

#### 2.2.2 Biochemical assay kits

The fungal melanin quantification kit and the total superoxide dismutase (SOD) and catalase (CAT) detection kits were purchased from HALING (Shanghai, China).

#### 2.2.3 Fluorescent probes

The reactive oxygen species (ROS) fluorescent probe was purchased from KeyGEN BioTECH (Nanjing, China). 4’,6-Diamidino-2-phenylindole (DAPI) for nuclear staining was obtained from Thermo Fisher Scientific.

### 2.3 Strain culture and preparation

*F. pedrosoi* clinical isolates used in this study were preserved in the Fungal Culture Collection of Shanghai Dermatology Hospital. After resuscitation, the strains were inoculated onto Sabouraud dextrose agar (SDA) and cultured at 28°C for 5–7 days or subcultured onto oatmeal agar (OA) to promote conidiation by optimizing nutritional conditions.

### 2.4 TCZ intervention experiments

Based on preliminary experiments, TCZ was added to SDA at final concentrations of 0, 5, 10, 25, 50, and 100 μg/mL. *F. pedrosoi* was inoculated into the drug-containing medium and cultured at 28°C for 7 days, after which colony color and morphological characteristics were recorded. After 30 days of culture, mycelia were collected and weighed to assess the effect of TCZ on fungal growth. Each experiment was independently repeated three times with three biological replicates per group.

### 2.5 Melanin extraction and quantification

*F. pedrosoi* was adjusted to 2 × 10^7^ colony-forming units (CFU)/well in 24-well plates and treated with or without 25 μg/mL TCZ for 10 days. Fungal melanin was extracted using the alkali-acid method described elsewhere (Potisek et al., 2021), and melanin content was quantified using a fungal melanin quantification kit.

### 2.6 SOD and CAT activity assays

SOD activity was measured using the nitroblue tetrazolium chloride photoreduction method, as modified from Beauchamp and Fridovich (Beauchamp and Fridovich, 1971). CAT activity was determined using the hydrogen peroxide decomposition method, following the protocol of Hadwan and Abed (Hadwan and Abed, 2016).

### 2.7 Phagocytosis assay

Phagocytosis of *F. pedrosoi* by human neutrophils was quantified using flow cytometry described by Hartung et al (Hartung et al., 2019). *F. pedrosoi* were labeled with FITC at 37°C for 30 min, washed, and adjusted to 3 × 10^6^ CFU/mL. Human neutrophils were divided into five groups (corresponding to **Figure 3A**): (1) NE group (1.5 × 10^6^ cells/mL neutrophils only); (2) NE + *Fp* group (neutrophils co-incubated with FITC-labeled *F. pedrosoi*); (3) NE + DHN-M group (neutrophils co-incubated with DHN-Melanin); (4) NE + TCZ group (neutrophils co-incubated with TCZ); (5) NE + *Fp* + TCZ group (neutrophils co-incubated with TCZ and FITC-labeled *F. pedrosoi*); All groups were incubated at 37°C (5% CO_2_) for 2 h. Samples were analyzed via BD FACSCelesta™. Phagocytosis rate (percentage of FITC^+^CD45^+^ neutrophils) was quantified using FlowJo 10.8.1 software.

### 2.8 NETosis immunofluorescence staining

To evaluate NETosis, human peripheral blood neutrophils (1.5 × 10^6^ cells/mL) were co-incubated with or without *F. pedrosoi* (3 × 10^6^ CFU/mL) for 24 hours with or without TCZ. Cells were fixed in 4% paraformaldehyde, permeabilized with 0.1% PBS-Triton for 5 min, and blocked with QuickBlock™ buffer for 10 min. Samples were incubated overnight at 4°C with anti-human MPO and anti-histone H3 antibodies (1:100 dilution), followed by incubation with goat anti-rabbit IgG secondary antibody (1:1000 dilution) for 1 hour at room temperature. Nuclear DNA was counterstained with DAPI, and images were captured using a fluorescence microscope. Fluorescence intensity was quantified using ImageJ by analyzing five random fields per sample. Integrated fluorescence density was measured after background subtraction.

### 2.9 Mouse infection model establishment

Twenty-five BALB/c mice were randomly assigned to five groups: (1) Blank control: injected with 40 μL of vehicle control solution; (2) Infection control: subcutaneously injected with 40 μL of *F. pedrosoi* suspension (3 × 10^7^ CFU/mL) into the right hind paw pad, followed by 40 μL of vehicle control solution 24 hours later; (3) DHN-melanin group: injected with 40 μL of DHN-melanin extracted from *F. pedrosoi* (3 × 10^7^ CFU/mL) into the right hind paw pad, followed by 40 μL of vehicle control solution 24 hours later; (4) TCZ intervention: injected with 40 μL of vehicle control solution, followed by an equal volume of TCZ (30 mg/kg) 24 hours later; (5) Infection + TCZ intervention: infected subcutaneously with 40 μL of *F. pedrosoi* and treated with TCZ (30 mg/kg) daily for 5 consecutive days starting 24 hours post-infection. The dose of 30 mg/kg was determined based on the results of this drug’s application in the pre-experimental animal model, which was established to evaluate drug tolerability and antifungal activity. On day 7, mice were euthanized using cervical dislocation, and the infected paw tissues were harvested for histopathological and immunofluorescence analysis.

### 2.10 Statistical analysis

All data were analyzed using GraphPad Prism 10.1. One-way analysis of variance (ANOVA) was used to assess statistical significance among multiple groups.

## 3 Results

### 3.1 Inhibitory effect of TCZ on colony morphology and growth of *F. pedrosoi*

**Figure 1:**
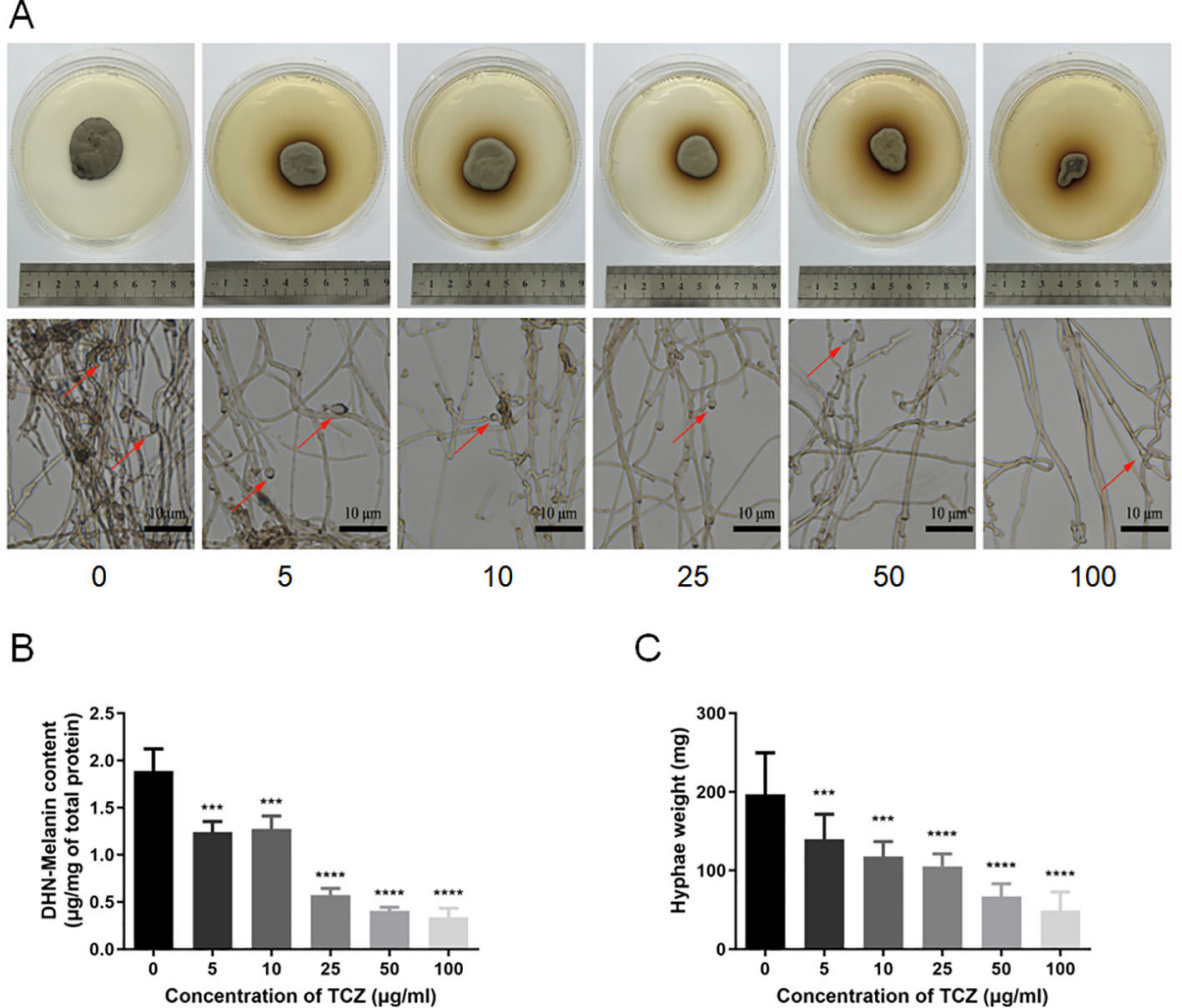
**(A)** Gross morphology of *F. pedrosoi* under different concentrations of TCZ; **(B)** DHN-melanin concentration in *F. pedrosoi* under different concentrations of TCZ (n=3); **(C)** Mycelial dry weight of *F. pedrosoi* under different concentrations of TCZ (n=3). Scale bar, 10μm. TCZ, tricyclazole; *F. pedrosoi*, *Fonsecaea pedrosoi*. ***p < 0.001, **** *p* < 0.0001.

To investigate the inhibitory effect of TCZ on *F. pedrosoi*, experimental groups with TCZ concentration gradients of 0, 5, 10, 25, 50, and 100 μg/mL were prepared using SDA plate medium, with the group lacking TCZ serving as the blank control. After 72 hours of incubation, dose-dependent changes were observed in *F. pedrosoi* colonies with increasing TCZ concentrations: colony diameter decreased, pigmentation significantly diminished, and colony color changed from dark black to grayish white (**Figure 1A**). Quantitative analysis of DHN-melanin content showed that DHN-melanin synthesis was significantly inhibited at TCZ concentrations ≥ 25 μg/mL (**Figure 1B**). Further measurement of mycelial dry weight revealed that TCZ concentrations ranging from 5 to 100 μg/mL significantly reduced the mycelial dry weight of *F. pedrosoi* compared to the control group (p < 0.05; **Figure 1C**). These results indicate that TCZ not only markedly alters the macroscopic characteristics of *F. pedrosoi* colonies but also exerts a strong inhibitory effect on fungal growth.

### 3.2 Regulation of TCZ on antioxidant enzyme system of *F. pedrosoi*

**Figure 2:**
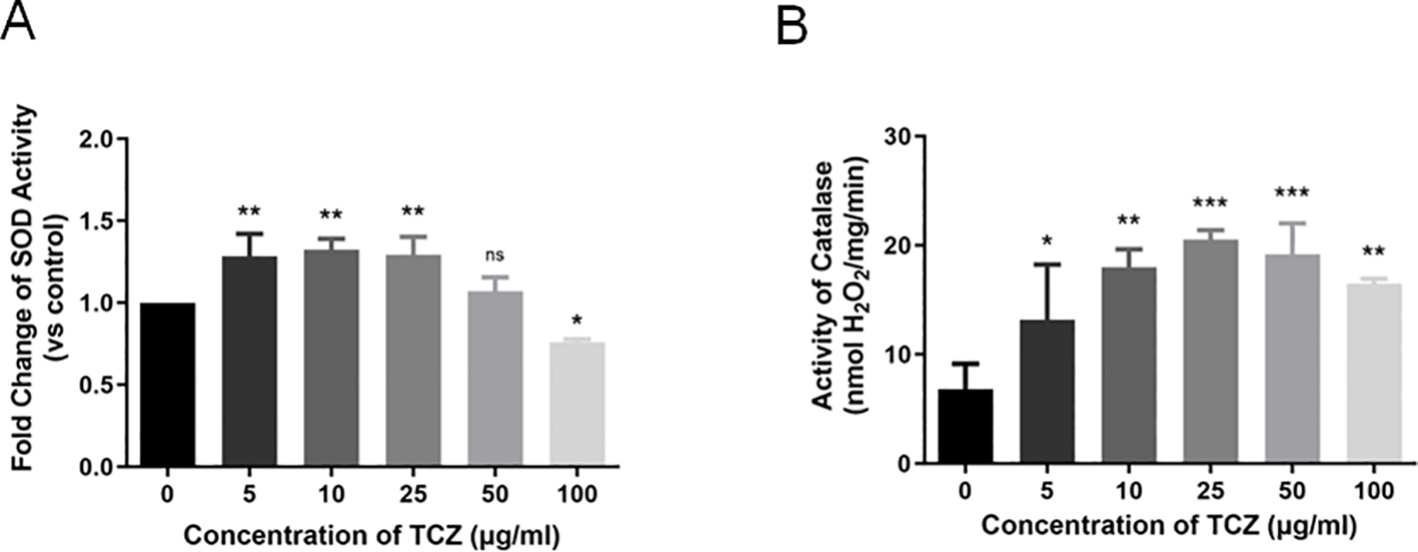
**(A)** SOD activity in *F. pedrosoi* under different concentrations of TCZ (n=3); **(B)** CAT activity in *F. pedrosoi* under different concentrations of TCZ (n=3). TCZ, tricyclazole; SOD, superoxide dismutase; CAT, catalase. *p < 0.05, **p < 0.01, ***p < 0.001.

Antioxidant enzyme activity serves as a reliable indicator of organismal stress responses. To further explore the mechanisms by which TCZ affects *F. pedrosoi* morphology, growth, and DHN-melanin synthesis, the antioxidant defense system of *F. pedrosoi* was analyzed. At lower TCZ concentrations (≤ 50 μg/mL), SOD activity in F. pedrosoi significantly increased, suggesting that the fungus responded to TCZ-induced oxidative stress by enhancing SOD activity (**Figure 2A**). However, at 100 μg/mL, SOD activity declined markedly to levels even lower than those of the untreated control. In contrast, CAT activity was significantly upregulated in all TCZ-treated groups (**Figure 2B**), with statistically significant differences compared to the control group (p < 0.05), indicating that CAT plays a consistent and central role in the TCZ-mediated inhibition of *F. pedrosoi*. Nevertheless, a slight reduction was observed at the highest concentration (100 μg/mL), although CAT activity remained above that of the untreated control. Collectively, these results suggest that TCZ disrupts the balance of the antioxidant enzyme system, intensifies endogenous oxidative stress, and thereby inhibits fungal growth.

### 3.3 TCZ potentiates human neutrophil oxidative stress capacity in *F. pedrosoi* infection

**Figure 3:**
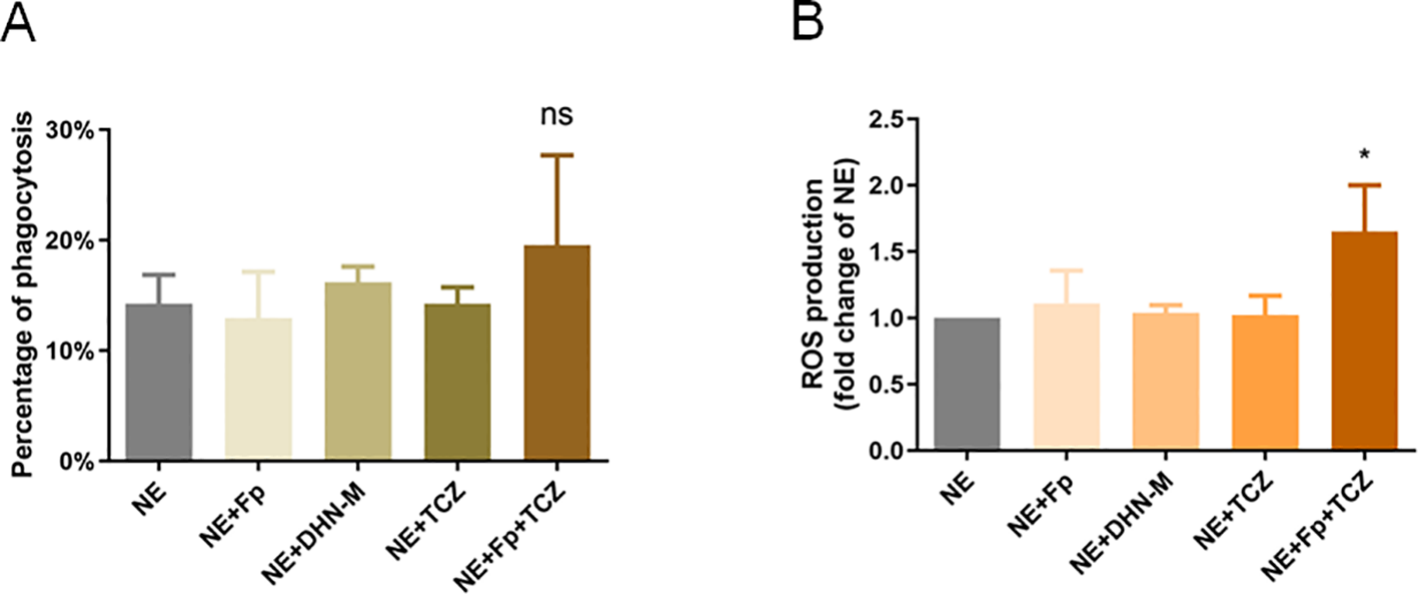
**(A)** Changes in neutrophil phagocytic function across experimental groups (n=3); **(B)** Changes in neutrophil reactive oxygen species (ROS) production across experimental groups (n=3). NE, neutrophils; *Fp*, *Fonsecaea pedrosoi*; DHN-M, 1,8-dihydroxynaphthalene-melanin; TCZ, tricyclazole. *p < 0.05.

Based on preliminary findings, it was hypothesized that the inhibitory effect of TCZ on *F. pedrosoi* involves interference with its oxidative stress response system. Neutrophils, as primary producers of ROS in host antifungal immunity, play a crucial role in maintaining oxidative balance. To elucidate the regulatory effect of TCZ on host immune cell function, multiple *in vitro* co-culture systems were established to evaluate its impact on human neutrophils’ antifungal activity. Using a flow cytometry-based approach, no statistically significant difference in neutrophil-associated *F. pedrosoi* signal was detected among the co-culture conditions tested (p > 0.05; **Figure 3A**). In contrast, TCZ treatment resulted in a significant increase in neutrophil-derived ROS levels compared with the control group (p < 0.05; **Figure 3B**).

### 3.4 TCZ promotes release of antimicrobial NETs in *F. pedrosoi*-infected human neutrophils

**Figure 4:**
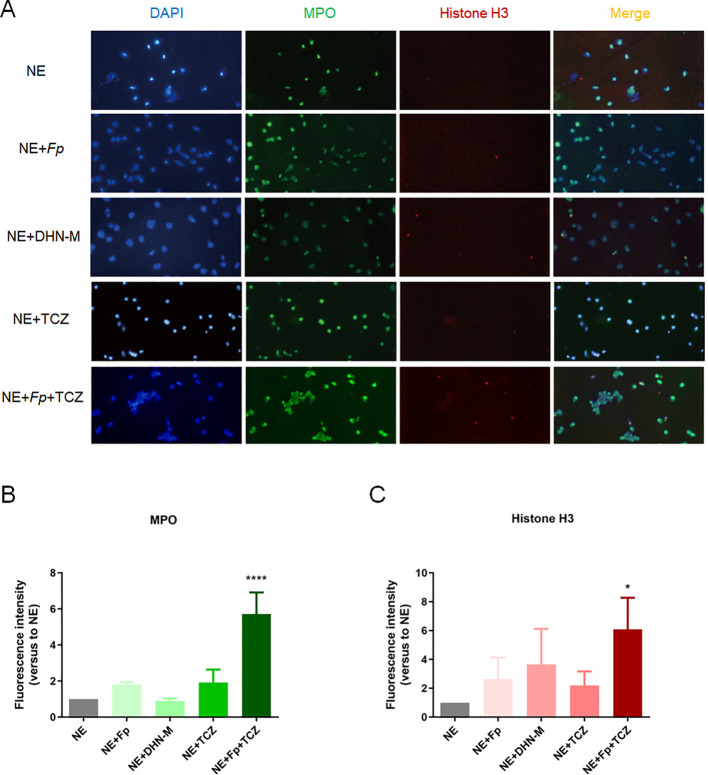
**(A)** NETs formation across experimental groups; **(B)** MPO expression across experimental groups (n=3); **(C)** Fluorescence intensity of histone H3 across experimental groups (n=3). Scale bar, 20μm. NETs, neutrophil extracellular traps; MPO, myeloperoxidase; NE, neutrophils; *Fp*, *Fonsecaea pedrosoi*; DHN-M, 1,8-dihydroxynaphthalene-melanin; TCZ, tricyclazole. *p < 0.05, **** *p* < 0.0001.

Neutrophils exert antimicrobial effects not only through ROS release but also via ROS-dependent NET formation. NETs consist of a DNA scaffold, histones (e.g., H3, H4), and antimicrobial proteins (e.g., MPO, neutrophil elastase [NE]) (Liu et al., 2025). To characterize neutrophil-derived NETs, immunofluorescence staining and quantitative analysis were conducted. As shown in **Figure 4A**, the fluorescence intensities of MPO and citrullinated histone H3, hallmark proteins of NETs, increased approximately 5-fold in the TCZ-treated co-culture group compared to the control group (p < 0.05; **Figures 4B, C**), clearly demonstrating enhanced NET production. These findings indicate that TCZ significantly boosts the antimicrobial function of human neutrophils during *F. pedrosoi* infection.

### 3.5 TCZ effectively inhibits neutrophil hyperactivation and alleviates inflammation in a CBM mouse model

**Figure 5:**
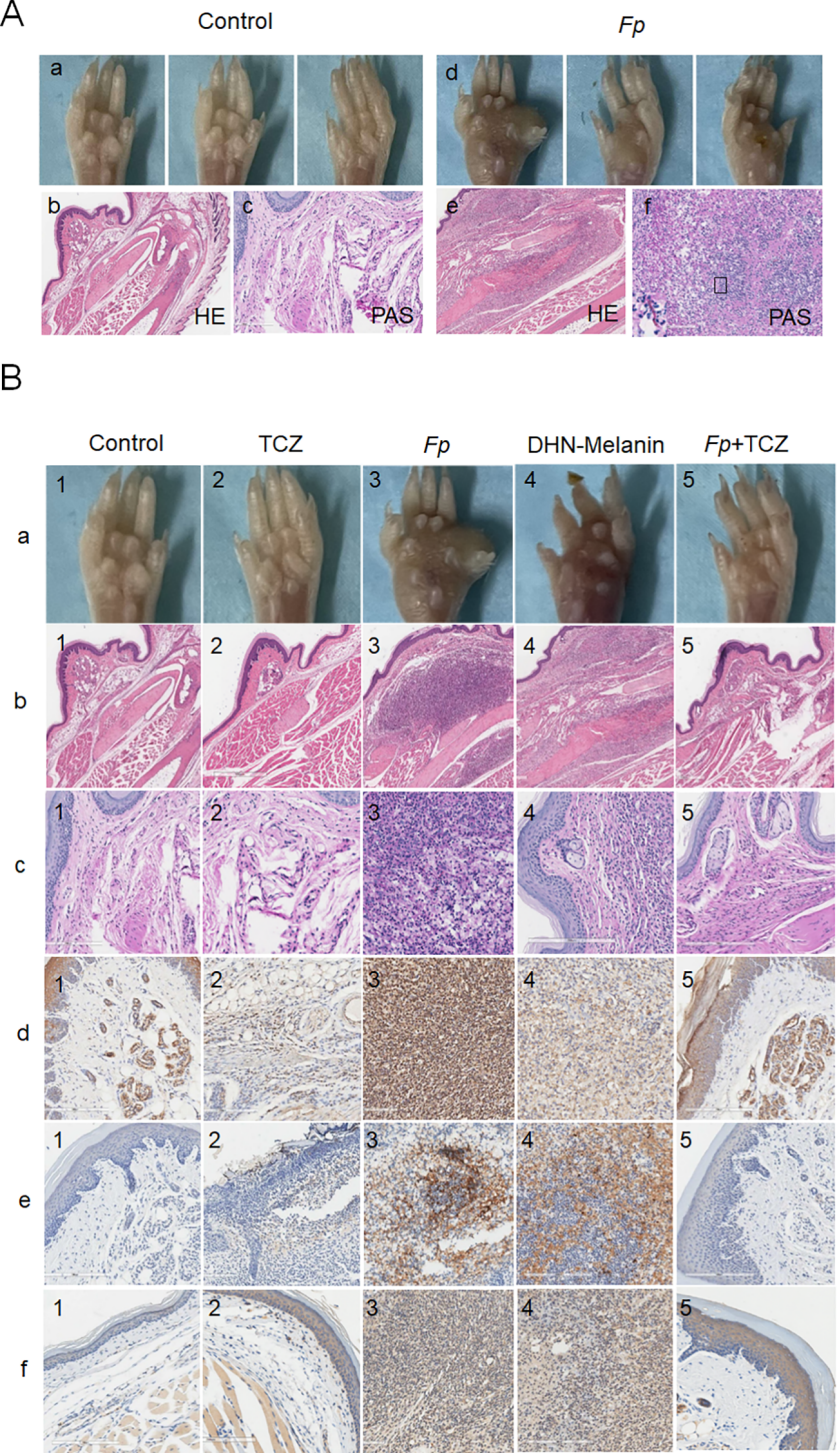
**(A)** (a) Phenotyping of mouse paw pads in control group mice; (b) HE staining in control group mice; (c) PAS staining in control group mice; (d) Phenotyping of mouse paw pads in experimental mice; (e) HE staining in experimental mice; (f) PAS staining in experimental mice. **(B)**: (a) Gross images of mouse paw pads across experimental groups; (b) HE staining of mouse paw pads across experimental groups; (c) PAS staining of mouse paw pads across experimental groups; (d) MPO staining of mouse paw pads across experimental groups; (e) Histone H3 staining of mouse paw pads across experimental groups; (f) Neutrophil staining of mouse paw pads across experimental groups. Scale bar, 20μm. HE, hematoxylin-eosin; PAS, periodic acid-Schiff; MPO, myeloperoxidase; NE, neutrophils; *Fp*, *Fonsecaea pedrosoi*; DHN-M, 1,8-dihydroxynaphthalene-melanin; TCZ, tricyclazole.

To validate *in vitro* findings, an *in vivo* CBM mouse model infected with *F. pedrosoi* was established. Successful model establishment was confirmed through paw pad phenotype observation and histopathological analyses, including hematoxylin-eosin (HE) and periodic acid-Schiff (PAS) staining. Paw pads of normal control mice appeared smooth, with uniform skin color and no pathological signs such as redness, swelling, or damage. In contrast, paw pads of *F. pedrosoi*-infected mice showed significant abnormalities, including swelling, thickened texture, dark discoloration, pigmentation, ulceration, and nodular protrusions. Histological examination revealed diffuse infiltration of inflammatory cells in the paw tissues of infected mice, indicating active and persistent local inflammation. PAS staining showed characteristically septate mycelia, primarily localized to regions of inflammatory cell infiltration or interstitial spaces, confirming colonization by *F. pedrosoi* (**Figure 5A**).

Following successful model validation, group intervention experiments were conducted to systematically evaluate the effects of TCZ on neutrophil function and inflammation. As shown in **Figure 5B**, compared to the normal control group, mice infected with *F. pedrosoi* or treated with isolated DHN-melanin displayed marked paw swelling, increased infiltration of neutrophils, and significantly elevated expression of neutrophil activation markers such as MPO and histone H3, indicating neutrophil hyperactivation. PAS staining further confirmed ongoing fungal infection in the *F. pedrosoi*-infected group. In striking contrast, the *F. pedrosoi*-infected group treated with TCZ alone showed a favorable therapeutic response: PAS staining revealed no fungal structures, MPO and histone H3 levels were not significantly different from those in the control group, and neutrophil infiltration was substantially reduced. These findings demonstrate that TCZ modulates neutrophil activity by inhibiting DHN-melanin synthesis, effectively controlling *F. pedrosoi* infection *in vivo* and mitigating associated inflammatory damage.

## 4 Discussion

The refractoriness and recurrence of CBM remain clinical challenges, closely associated with fungal evasion of the host immune system, which drives disease chronicity (Queiroz-Telles et al., 2017). Melanin, a virulence factor of *F. pedrosoi*, may play a key role in disease persistence. Although the melanin inhibitor TCZ has previously been used in agricultural and *in vitro* studies of CBM (Franzen et al., 2006; Cunha et al., 2010; Heidrich et al., 2021; Pham et al., 2024), its effects on neutrophil functions remain unclear, and the significance of neutrophil-mediated fungal control is still incompletely understood.

Our results demonstrated that TCZ significantly reduced DHN-melanin synthesis in *F. pedrosoi*, leading to lighter colony pigmentation and decreased hyphal biomass, consistent with previous findings from melanin-targeted antifungal studies (Cunha et al., 2005; Franzen et al., 2006; Kumar et al., 2015; Kong et al., 2018; Koehler et al., 2021). The oxidative stress response represents a critical host defense against fungal pathogens. Our findings indicate that TCZ disrupts the fungal antioxidant enzyme system in a concentration-dependent manner. At lower concentrations (≤50 μg/mL), TCZ induced a compensatory antioxidant response, as evidenced by the increased SOD activity—likely reflecting the fungal attempt to counteract moderate oxidative stress. However, at 100 μg/mL, SOD activity markedly decreased to levels even lower than the untreated control, suggesting loss of structural integrity or oxidative inactivation of the enzyme under excessive stress (Herrero et al., 2008; Cunha et al., 2010; Yaakoub et al., 2022). Similar concentration-dependent oxidative inactivation of antioxidant enzymes has been reported in fungal systems exposed to high ROS burdens (e.g., DHN-melanin deficiency–related oxidative collapse) (Cunha et al., 2010). CAT activity exhibited a different pattern: it was elevated across all TCZ concentrations but showed a slight decline at the highest concentration (100 μg/mL), though still above baseline. This may indicate that CAT expression and enzymatic turnover reached their physiological upper limit, followed by stress-induced overload. Previous biochemical studies have demonstrated that excessive hydrogen peroxide can inhibit CAT activity through oxidative modification or structural damage to the enzyme (Ransy et al., 2020). Taken together, these observations suggest that high-dose TCZ shifts the antioxidant response from a compensatory activation phase to a dysfunctional or inhibitory phase. The divergent trends of SOD and CAT imply a breakdown of the antioxidant cascade, leading to uncontrolled intracellular ROS accumulation and compromising fungal viability. This dynamic imbalance aligns with prior reports showing that fungi with impaired oxidative defenses rapidly lose survival capacity under ROS stress (Ren et al., 2012; Yaakoub et al., 2022).

Neutrophils are pivotal effectors in CBM infection, yet their antimicrobial functions are profoundly suppressed by DHN-melanin. Previous studies have shown that neutrophils exert fungicidal effects on *F. pedrosoi* conidia through ROS generation (Breda et al., 2020a). Our results revealed that human neutrophils’ ROS production was significantly increased following TCZ treatment, indicating enhanced fungicidal activity. Using an exploratory flow cytometry-based approach, no statistically significant increase in neutrophil-associated *F. pedrosoi* signal was observed under the tested conditions. Previous literature suggests that neutrophil-mediated fungal killing is not exclusively dependent on phagocytosis. Phagocytes can release extracellular ROS through non-phagocytic pathways, which contribute to the killing of extracellular fungal elements, including hyphae and conidia (Bylund et al., 2010). Beyond direct oxidative damage (Nguyen et al., 2017), ROS also serve as key signaling molecules in NETosis formation. The two primary sources of ROS in neutrophils are NADPH oxidase and mitochondria, with NET release being primarily dependent on NADPH oxidase-derived ROS (Brinkmann et al., 2004). As a specialized neutrophil defense mechanism, NETosis—characterized by the release of MPO and citrullinated histone H3—was significantly enhanced in the TCZ-treated group, consistent with the improved fungicidal capacity of NETs. Collectively, these data suggest that DHN-melanin suppresses NETosis by masking fungal cell wall antigens or neutralizing ROS, while TCZ alleviates this suppression through melanin depletion, thereby restoring neutrophil effector functions.

*In vivo* experiments were conducted to evaluate the effects of TCZ treatment in a murine CBM infection model. Our data demonstrated that TCZ significantly alleviated paw pad swelling, reduced inflammatory cell infiltration, and inhibited neutrophil hyperactivation (as evidenced by decreased MPO and histone H3 expression), with no detectable fungal colonization. These findings align with the *in vitro* results, indicating that TCZ effectively controls fungal infection by inhibiting DHN-melanin synthesis and activating neutrophil functions. Notably, melanin injection alone induced neutrophil activation, suggesting that melanin may act as a damage-associated molecular pattern to trigger immune responses (Zhang et al., 2025), although excessive activation may lead to tissue damage. The amelioration observed following TCZ treatment warrants further investigation to determine whether it is mediated by the immunomodulatory effects of TCZ.

Current CBM treatment relies on long-term antifungal therapies, but the disease remains prone to recurrence. Previous studies have shown that TCZ holds unique value in CBM management; *in vitro* susceptibility testing indicates that TCZ significantly enhances the efficacy of first-line antifungals such as itraconazole and terbinafine against *F. pedrosoi* (Heidrich et al., 2021). The present study further confirms TCZ’s multifaceted activity: it inhibits *F. pedrosoi* proliferation by reducing DHN-melanin synthesis and disrupting the fungal antioxidant system, while simultaneously enhancing neutrophil-mediated oxidative killing via increased ROS production and NETosis, without affecting phagocytic efficiency. These effects collectively contribute to reduced disease severity in the murine CBM model. Altogether, our findings support the use of TCZ as a promising adjuvant therapeutic agent. Optimizing TCZ’s dosing regimen, including dose, concentration, and treatment duration, and selecting appropriate administration methods (e.g., topical versus injectable) will be essential for maximizing its antifungal synergy while minimizing the systemic toxicity associated with long-term antifungal use. Moreover, validating its efficacy against other CBM pathogens such as *Cladophialophora carrionii* could broaden its clinical applicability and potentially redefine current treatment standards. Through its synergistic enhancement of antifungal efficacy and modulation of host immunity, TCZ-based combination therapies have the potential to overcome the refractory nature and high recurrence rate of CBM, offering a more sustainable strategy for managing difficult infections and ultimately improving clinical outcomes and prognosis. These findings align with the unmet need for novel therapeutic approaches in addressing the challenges of CBM treatment.

The clinical safety of TCZ requires thorough evaluation. Previous studies have established a favorable safety profile, supporting its potential clinical use (Corvaro and Bartels, 2019). Oral pharmacokinetic studies in rodents have shown that TCZ has high bioavailability (> 86%), rapid absorption and distribution to major organs, extensive hepatic metabolism (producing approximately 30 metabolites), and rapid excretion through both urinary (31–64%) and fecal (39–65%) routes, with no evidence of bioaccumulation (Corvaro and Bartels, 2019). Furthermore, *in vitro* studies confirm that human liver microsomes do not generate unique metabolites, indicating metabolic similarity between rodents and humans and supporting the translational relevance of preclinical data (Corvaro and Bartels, 2019). For the proposed topical clinical application, systemic exposure is expected to be much lower than that associated with oral dosing. Given that oral doses used in rodent studies are approximately 10,000-fold higher than estimated human exposure (Corvaro and Bartels, 2019), systemic toxicity from topical application should be minimal. Nonetheless, further assessments of local toxicity and potential irritant responses are warranted, and the development of low-toxicity formulations, suitable administration routes, and optimized concentration regimens will be crucial to advancing the clinical use of TCZ as an adjuvant therapy.

This study has several limitations. First, the molecular mechanisms by which TCZ modulates neutrophil function, including the signaling pathways involved in NADPH oxidase activation and NET release, and how TCZ reverses DHN-melanin-mediated inhibition, remain unclear. Second, dynamic changes in key cytokines (e.g., IFN-γ, IL-17), which are essential for neutrophil regulation, were not comprehensively evaluated. Third, findings from *in vitro* and murine models require validation in clinical samples to account for potential species-specific differences. Clinically, TCZ’s potential as a topical adjuvant for dematiaceous fungal infections warrants investigation, including the development of advanced drug delivery systems (e.g., local pump infusion) to enhance lesion-specific drug absorption while reducing systemic side effects. In summary, future research should proceed along three dimensions: elucidating molecular mechanisms, validating findings in clinical samples, and optimizing drug delivery systems. Together, these efforts will establish a stronger theoretical and practical foundation for the application of TCZ as an adjuvant therapy for CBM, potentially transforming the role of agricultural chemical compounds in clinical medicine.

## 5 Conclusion

This study reveals a novel mechanism by which TCZ combats *F. pedrosoi* infection, offering a strategy of melanin synthesis inhibition and immune function activation for CBM treatment. Future research will focus on elucidating the molecular mechanisms underlying TCZ-mediated regulation of neutrophils and evaluating cytokine dynamics. These endeavors are intended to better explore the potential of TCZ as an adjuvant therapeutic strategy for CBM, ultimately laying a foundation for the development of TCZ into a novel antifungal therapy. 

## Data Availability

The raw data supporting the conclusions of this article will be made available by the authors, without undue reservation.
